# Diverging contaminant profiles and prokaryotic assemblages in Arctic and Antarctic lake sediments

**DOI:** 10.3389/fmicb.2025.1722478

**Published:** 2026-01-21

**Authors:** Alessandro Ciro Rappazzo, Angelina Lo Giudice, Stefania Giannarelli, Carmen Rizzo, Alessio Tomei, Lisa Ghezzi, Warren R. L. Cairns, Maurizio Azzaro, Maria Papale

**Affiliations:** 1 Institute of Polar Sciences, National Research Council (CNR-ISP), Messina, Italy; 2 Department of Chemistry and Industrial Chemistry, University of Pisa, Pisa, Italy; 3 Stazione Zoologica Anton Dohrn, Department Marine Biotechnology, National Institute of Biology, Messina, Italy; 4 Department of Earth Sciences, University of Pisa, Pisa, Italy; 5 Institute of Polar Sciences, National Research Council (CNR-ISP), Venice, Italy; 6 Department of Environmental Sciences, Informatics and Statistics, Ca’ Foscari University of Venice, Venice, Italy

**Keywords:** Antarctica, Arctic, bacterial communities, biodiversity, contamination, heavy metals, persistent organic pollutants, polar lakes

## Abstract

**Introduction:**

Persistent organic pollutants (POPs) and trace metals are increasingly recognized as critical drivers of ecological change in polar environments. However, their combined impact on sediment microbial communities remains largely unexplored.

**Methods:**

We analyzed sediments from 12 high-latitude lakes and ponds, five from the Arctic (Svalbard) and seven from the Antarctic (South Shetland Islands/Deception Island), to examine contaminant profiles (polychlorinated biphenyls [PCBs] and trace metals) and prokaryotic community structure using 16S rRNA gene amplicon sequencing. Finally, we assessed the associations between the identified communities and detected pollutants, and compared these associations across lakes and sites.

**Results:**

The results revealed distinct chemical signatures between poles: Arctic sediments were mainly contaminated by polycyclic aromatic hydrocarbons (∑PAHs, 18.5–685.7 ppb; phenanthrene was the most abundant), whereas Antarctic sediments showed relatively higher concentrations of chlorobenzenes (∑CBs, 1.9–3.6 ppb) and polychlorinated biphenyls (∑PCBs, 0.9–1.4 ppb), with 2-methylnaphthalene as the most abundant PAH. Manganese was the most abundant metal in both regions, reaching 760 ppm in the Arctic, while elevated arsenic and lead characterized specific Antarctic sites. Amplicon sequencing identified five dominant phyla (i.e., Actinobacteriota, Bacteroidota, Alpha- and Gammaproteobacteria, and Desulfobacterota) with significant compositional shifts between poles.

**Discussion:**

Notably, the distinct contaminant signatures between regions appeared to be associated with shifts in microbial community composition, suggesting that both the type and intensity of POP and metal exposure may influence bacterial diversity and ecological functions in polar lake sediments. These findings provide a robust baseline for Arctic–Antarctic comparisons, positioning polar lakes as sensitive sentinels of contaminant-driven ecological change. They also underscore the urgent need for functional studies and long-term monitoring to evaluate ecosystem resilience under accelerating climate change.

## 1 Introduction

Lakes and ponds are prominent features of the Arctic landscape and are similarly present across many areas of Antarctica. These aquatic environments in polar regions often represent transitory habitats that can undergo rapid changes, from their initial creation to the eventual filling of the basin with abiotic and biotic sediments, or even catastrophic draining when ice-dammed valleys are lost during glacial melt (Vincent et al., 2009).

High-latitude lakes are of significant global importance, serving as habitats for unique species and communities and acting as sentinels of environmental change (Saros et al., 2022; Calizza et al., 2022). Variations in size, number of freeze-thaw cycles, nutrient availability, local food webs, and mineral leaching contribute to the considerable seasonal fluctuations of these habitats, affecting the distribution and composition of their microbial communities (Izaguirre et al., 1998; Vinocur and Unrein, 2000; Borghini et al., 2011; Archer et al., 2015; Seppey et al., 2023). This makes polar lakes and ponds invaluable natural laboratories for studying the biogeographical distribution and adaptation of microbial communities, particularly in the context of contaminant exposure (Seppey et al., 2023).

Microbial communities in these extreme environments often display unique physiological adaptations and metabolic pathways, enabling them to thrive under harsh conditions (Kiersztyn et al., 2019) and drive essential biogeochemical cycles for ecosystem functioning (Zhang et al., 2025). The increasing presence of both organic and inorganic contaminants in these pristine environments is a growing concern, as these pollutants can significantly alter the composition and function of prokaryotic communities, thereby impacting nutrient cycling and biogeochemical processes (Custodio et al., 2022).

Direct and indirect inputs of man-made pollutants, originating from local emissions and remote continental sources, lead to both the Arctic and Antarctica as ultimate sinks for these substances. This can result in unexpected disturbances and pose risks to polar ecosystems, creating a global issue for the Earth in the future (AMAP, 2017; AMAP, 2021). Even minor alterations in their physical, chemical, or biological traits can trigger substantial changes in their limnological characteristics and the overall structure and function of the ecosystem (Quayle et al., 2002; Flocco et al., 2019; Rosa, 2019; Ogaki et al., 2020). Within these systems, sediments play a critical role, acting both as long-term sinks for organic and inorganic contaminants and as archives of ecological change (Chiaia-Hernández et al., 2022). By integrating inputs from the water column and catchment, sediments accumulate and preserve pollutants, while simultaneously hosting diverse microbial communities that mediate contaminant transformation and drive biogeochemical cycling (Chen et al., 2019; Chiaia-Hernández et al., 2022; Lyautey et al., 2021). This dual function makes microbial assemblages in sediment sensitive indicators of anthropogenic pressures in polar lakes. This investigation specifically targets legacy and priority organic contaminants, placing particular emphasis on four classes of persistent organic pollutants: polycyclic aromatic hydrocarbons (PAHs), polychlorinated biphenyls (PCBs), polychloronaphthalenes (PCNs), and chlorobenzenes (CBs). This selection establishes a multifaceted diagnostic framework for polar lake sediments, encompassing: globally regulated legacy POPs characterized by well-established long-range transport (Zhang et al., 2024); combustion- and fuel-derived priority pollutants that exhibit strong partitioning into sediments and are extensively documented in Arctic contexts (Simon et al., 2023); under-monitored yet ecologically pertinent chlorinated aromatics whose presence in high-latitude environments is gaining increased recognition (Muir et al., 2025); and chlorinated industrial compounds that indicate both hemispheric transport and proximate inputs from nearby research stations and coastal infrastructure (Publications Office of the European Union, 2020; Vorkamp et al., 2022). Understanding these impacts is crucial for assessing the ecological health of polar aquatic ecosystems and for predicting their resilience to anthropogenic pressures (Vázquez et al., 2017). This study aimed to assess the concentrations of POPs and trace metals in polar lake sediments and to characterize their associated bacterial communities. The experimental plan was also designed to identify potential links between contaminant loads and microbial assemblage composition by comparing lakes from the Arctic (Svalbard) and Antarctica (South Shetland Islands/Deception Island), thereby providing baseline data for future evaluations of ecosystem responses to environmental change.

## 2 Materials and methods

### 2.1 Sampling area and sample collection

**Figure 1:**
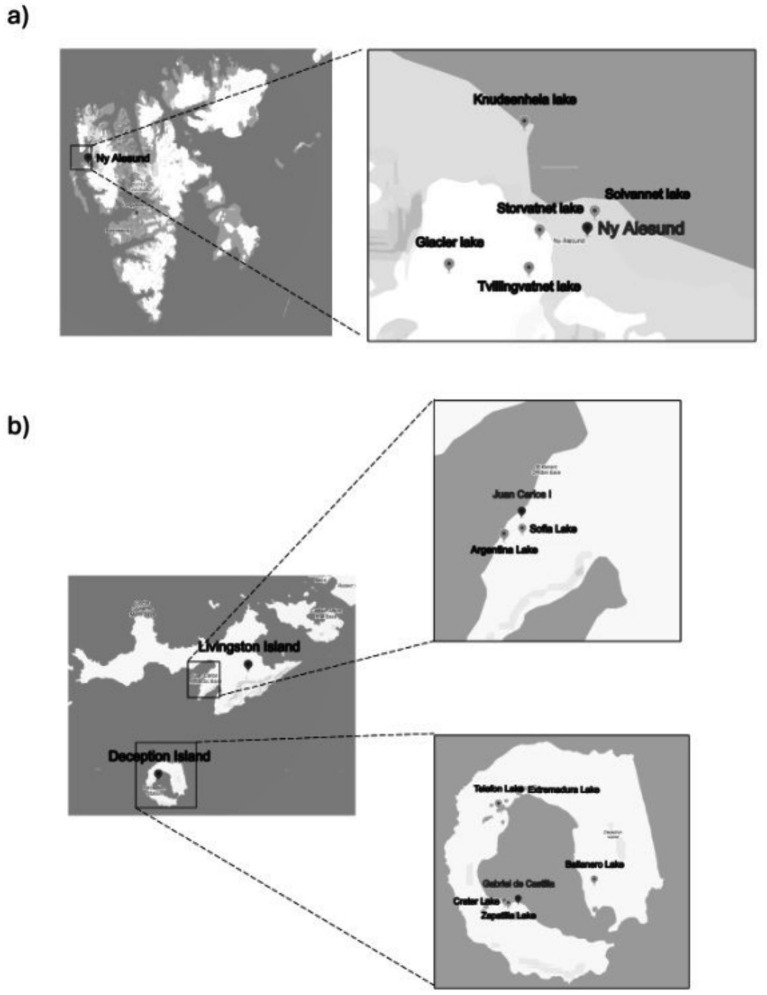
Maps showing the sampling areas. **(a)** Lakes lying in the Ny-Ålesund area (Svalbard Islands, High Arctic Norway). **(b)** Lakes lying in Deception Island and Livingston Island (Antarctica).

Two sampling campaigns were conducted: the first in Ny-Ålesund (Svalbard Islands, High Arctic Norway), from August 5th to 18th, 2021, and the second in the South Shetland Islands archipelago, Antarctica, from January 25th to February 1st, 2022. Sediment samples were collected from a total of 12 lakes, five lakes surrounding the Ny-Ålesund research village: Solvannet (L1), Glacier (L2), Knudsenheia (L3), Storvatnet (L4), and Tvillingvatnet (L5) (Figure 1a); and seven from Antarctica: Argentina (LA) and Sofia (LS) both in Livingston Island, and Ballaneros (LB), Crater (LC), Extremadura (LE), Telefon (LT) and Zapatilla (LZ) (all in Deception Island) (Figure 1b). Based on their characteristics, lakes were classified in different categories: brackish (L3, LT, LB, LE), glacial (L2, LS), anthropogenic or animals impacted (L1, L4, LA, LC) and water supply lakes (L5 and LZ) (Table 1). The uppermost 10 cm of sediment were manually collected from each lake at a depth of 30–60 cm. For microbiological analyses, samples (approx. 25 g) were collected using a pre-sterilized bailer and transferred into sterile plastic containers. Concurrently, pre-cleaned plastic tanks were employed for the collection of samples (approx. 50 g) for trace metal analysis, whereas pre-cleaned aluminum containers were used for samples (approx. 50 g) destined for POPs determination. Samples were subsequently transported to the research stations’ laboratories for immediate storage at −20 °C until further processing.

**Table 1:** Selected lakes’ sediment coordinates and categories of analysed sediment samples.

Regions	Area	Lake	Sample ID	Coordinates		Categories
Arctic	Ny-Ålesund	Solvannet	L1	N 78°55.552′	E 11°56.327′	Anthropogenic/animals impacted
		Glacier	L2	N 78°55.044′	E 11°47.442′	Glacial
		Knudsenheia	L3	N 78°56.680′	E 11°51.579′	Brackish
		Storvatnet	L4	N 78°55.453′	E 11°52.728′	Anthropogenic/animals impacted
		Tvillingvatnet	L5	N 78°55.058′	E 11°51.922′	Base water supply
Antarctic	Livingston Island	Sofia	LS	S 62°40′12.19″	W 60°23′17.90″	Glacial
		Argentina	LA	S 62°40′22.39″	W 60°24′18.12″	Anthropogenic/animals impacted
	Deception Island	Crater	LC	S 62°59′00.68″	W 60°40′20.41″	Anthropogenic/animals impacted
		Zapatilla	LZ	S 62°59′00.24″	W 60°40′29.07″	Base water supply
		Extremadura	LE	S 62°55′12.2″	W 60°39′47.0″	Brackish
		Telefon	LT	S 62°55′39.9″	W 60°41′21.3″	Brackish
		Ballaneros	LB	S 62°58′51.1″	W 60°34′27.1″	Brackish

### 2.2 Taxonomic composition of the bacterial communities

#### 2.2.1 Total DNA extraction and NGS target sequencing

Total DNA was extracted from environmental samples using the DNeasy^®^ PowerSoil^®^ Pro Kit (Qiagen) according to the manufacturer’s instructions. The genomic DNA obtained was resuspended in 100 μL of Milli-Q water and stored frozen at −20 °C for subsequent analyses. DNA concentrations and purity were quantified by NanoDrop ND-1000 UV–Vis spectrophotometer (NanoDrop Technologies, United States). The bacterial 16S rDNA region V3–V4 was amplified using universal primers 341F (5′-CCTACGGGNGGCWGCAG-3′) and 785R (5′-GACTACHVGGGTATCTAATCC-3′) (Abellan-Schneyder et al., 2021). Sequencing was performed on the Illumina MiSeq platform, following the standard protocols of Eurofins Europe Services (Germany).

#### 2.2.2 Bioinformatics analysis

Raw paired-end reads were processed in R (v. 4.4.2) using the DADA2 pipeline (Callahan et al., 2016) to obtain amplicon sequence variants (ASVs). Forward and reverse reads were first inspected by per-base quality profiles and then filtered and trimmed with filterAndTrim, removing reads with ambiguous bases (maxN = 0), enforcing expected error thresholds (maxEE = 2 for both directions), truncating low-quality tails, and trimming a short number of bases to exclude primer remnants and low-quality regions. Error rates for forward and reverse reads were learned using learnErrors. Dereplication was performed with derepFastq for each sample, and denoising was carried out separately on forward and reverse reads with the dada function using a pseudo-pooling approach (pool = “pseudo”). Denoised forward and reverse reads were merged with mergePairs, requiring a minimum overlap and allowing at most one mismatch in the overlap region. Merged sequences were then assembled into a sequence table with makeSequenceTable. Chimeric sequences were identified and removed *de novo* using removeBimeraDenovo in “consensus” mode, yielding a non-chimeric ASV table (seqtab.nochim). Taxonomic assignment of ASVs was performed with DADA2’s naive Bayesian classifier (assignTaxonomy), using the SILVA SSU for 16S rRNA genes (Quast et al., 2013), followed by species-level assignment with addSpecies when possible. Before phylogenetic reconstruction, we applied an additional filtering step to reduce noise and computational load. ASVs with very low total abundance across all samples (e.g., <10 reads) were removed from the sequence table, and putative non-target sequences were excluded based on their taxonomic assignment (e.g., chloroplasts, mitochondria, and Eukaryota). The remaining ASV sequences were then aligned using a multiple-sequence alignment algorithm implemented in the msa package (Bodenhofer et al., 2015), and a phylogenetic tree was inferred using phangorn (Schliep, 2011). Distances were computed under a maximum-likelihood framework, an initial neighbour-joining tree was constructed, and model parameters were optimised under a GTR + Γ + I model to obtain the final tree. Finally, the non-chimeric ASV table, taxonomic assignments, sample metadata, and phylogenetic tree were integrated into a single phyloseq object (McMurdie and Holmes, 2013). This object was used for all subsequent analyses of alpha- and beta-diversity, taxonomic composition (relative-abundance barplots), and ordination (nMDS, PCA, and related multivariate analyses).

### 2.3 Persistent organic pollutants (POPs)

Among POPs, these four groups (PCBs, PAHs, PCNs, CBs) cover: globally regulated legacy POPs (PCBs), combustion-derived priority pollutants with high sediment affinity (PAHs), lesser-monitored but relevant chlorinated aromatics (PCNs), and site-specific industrial contaminants (chlorobenzenes). This mix thus gives both a regulatory context and a diagnostic picture of historical vs. ongoing sources.

#### 2.3.1 Chemicals

n-Hexane, isooctane, acetone, and 2-propanol (pesticide grade) were obtained from Romil, Baker Analyzed, Carlo Erba, and Sigma-Aldrich, respectively. Certified standard solutions of polycyclic aromatic hydrocarbons (PAHs; L429-PAR) and polychlorinated biphenyls (PCBs; PCB-PAR-H) were purchased from Wellington Laboratories (United States). Prior to extraction, two different solutions containing ^13^C- and D-labelled analytes (Wellington Laboratories, United States) were added to evaluate extraction efficiency (PAHs: L429-IS; PCBs: MBP-MXE). Additional isotopically labelled standards were employed to monitor instrument performance during analysis (PAHs: L429-AS, L429-RS; PCBs: MBP-MXF). Solution MO040 (DTO Laboratories) contained chlorobenzenes, while MCBS (Wellington Laboratories) included labelled chlorobenzenes. Standards PCN-MXA and MXC (Wellington Laboratories) were used for polychloronaphthalene analysis.

Calibration solutions were prepared by diluting certified standards in isooctane. Sample preparation was carried out using a Bandelin ultrasonic bath (Sonorex super 10p), a Hettich centrifuge (Rotofix 32 A), a Jouan centrifugal evaporator (RC10.22 Sensitive Bio), a VELP Scientific magnetic stirrer, and an analytical balance (Als 220-4).

#### 2.3.2 Determination of persistent organic pollutants

Gas chromatographic analyses were performed using a GC-MS/MS system (Agilent 7890B GC coupled to a 7010 triple quadrupole MS, Agilent) equipped with an ALS autosampler. A DB-5MS UI capillary column (Agilent; 30 m × 0.25 mm i.d., 0.25 μm film thickness) was employed. Sample injections (1 μL) were carried out in pulsed splitless mode (60 psi, injector temperature 280 °C, solvent delay 6 min). Helium was used as the carrier gas (1.2 mL/min). The oven temperature program was set as follows: 40 °C (6 min), ramp at 20 °C/min to 55 °C, 50 °C/min to 120 °C, 20 °C/min to 200 °C, and 8 °C/min to 310 °C (hold for 3 min). The transfer line was maintained at 300 °C.

Quantification and confirmation were performed in dynamic multiple reaction monitoring (dMRM) mode, with optimized transitions for both quantification and analyte identification. Calibration was based on native and isotopically labelled standards for PAHs, PCBs, CBs, and PCNs (Wellington Laboratories and DTO Laboratories). Target analyte concentrations were determined by correcting peak areas relative to internal injection standards. Blank signals were subtracted, and concentrations were further adjusted for recovery using the appropriate isotopically labelled method standards.

#### 2.3.3 Determination of trace metals

Sediments were dissolved in reversed aqua regia using the Ethos Easy microwave platform (Milestone Srl, Sorisole, Italy). About 500 mg of homogenized sample were placed in a fluorocarbon polymer microwave vessel with 7.5 mL of concentrated HNO3 and 2.5 mL of concentrated HCl (super pure-grade acids, Romill-SpA). Before analysis the samples were filtered with 0.45 μm syringe filter and opportunely diluted. Trace elements were determined using inductively coupled plasma mass spectrometry (ICP-MS; Agilent 7850, Santa Clara, CA, United States). Analytical uncertainty was evaluated by the analysis of soil reference material NIST 2711a (Montana soil) and ERM-CC018 (sandy soil). RSD was within 5%, and the accuracy, defined as the recovery percentage, was between 63 and 120% for NIST 2711a and between 93 and 106% for BAM ERM-CC018. The certified values in NIST 2711a are reported as total content in the sample, whereas the certified values in BAM ERMcc018 are reported as aqua regia extractable content.

### 2.4 Statistical analysis

Statistical analyses were performed in R software (v. 4.4.2). Alpha diversity indices (Observed ASVs/richness, Shannon and Simpson) were calculated from the ASV count table. Beta diversity was evaluated using Bray–Curtis dissimilarities computed on relative-abundance-transformed ASVs. Community patterns were visualised using non-metric multidimensional scaling (nMDS) based on Bray–Curtis distances, implemented in the vegan package (Oksanen et al., 2022). Group differences in community composition by region and lake category were tested using PERMANOVA (adonis2, vegan) with 9,999 permutations. To verify that significant PERMANOVA outcomes were not driven by heterogeneity in multivariate dispersion, homogeneity of dispersion among groups was assessed with betadisper and associated permutation tests. To characterize environmental and contaminant gradients, principal component analysis (PCA) was applied to the chemical datasets using the stats and factoextra packages (Kassambara and Mundt, 2020). Separate PCAs were performed on (i) persistent organic pollutants (ΣPAHs, ΣPCBs, ΣCBs, ΣPCNs) and (ii) trace elements (Li, Be, V, Cr, Mn, Co, Ni, Cu, Zn, As, Se, Sr, Mo, Cd, Sb, Ba, Tl, Pb, U). In addition, a third PCA biplot was constructed by combining environmental/chemical variables with observed ASVs to jointly visualise sample ordination and the association of diversity patterns with the main physicochemical and contaminant gradients. Pairwise associations between bacterial community structure and environmental chemistry were explored by Spearman rank correlation analyses between the relative abundances of the most abundant bacterial families and the complete set of environmental, POP, and trace-metal variables, using Spearman rank correlations implemented in the stats package; resulting *p*-values were adjusted for multiple comparisons using the Benjamini–Hochberg FDR.

## 3 Results

### 3.1 Taxonomic composition of the bacterial communities

Table 2 presents the total sequence reads, represented as ASVs, number retrieved for each lake sediment sample. For Arctic samples, a total of 416,463 reads were detected, of which 356,127 were of good quality (85.51%). Instead, for Antarctic samples, 732,657 reads were detected, of which 640,187 were of good quality (87.38%). Unfortunately, the microbial community composition of Lake Glacier (L2) could not be evaluated because the sample tank failed during transport from the Arctic to the Italian laboratories, resulting in sample contamination (the complete ASV table is provided in Supplementary Table S1).

**Table 2:** Sequencing quality and diversity statistics for all sampling sites.

Sample ID	Lake	Reads in	Reads out	Trimming %	Merged	Merged %	Good quality reads	Good quality reads %	ASVs number
L1	Solvannet	104,422	99,298	95.09	97,647	93.51	89,188	85.41	1,192
L3	Knudsenheia	103,725	98,094	94.57	96,362	92.90	93,322	89.97	1,260
L4	Storvatnet	106,172	100,171	94.35	97,170	91.52	90,444	85.19	1,766
L5	Tvillingvatnet	102,144	95,753	93.74	92,720	90.77	83,173	81.43	1,874
LS	Sofia	100,640	94,756	94.15	94,200	93.60	89,071	88.50	456
LA	Argentina	109,229	103,666	94.91	102,356	93.71	96,470	88.32	1,297
LB	Ballaneros	119,498	113,433	94.92	111,028	92.91	100,701	84.27	1,723
LC	Crater	82,434	78,425	95.14	77,349	93.83	74,163	89.97	1,159
LE	Extremadura	127,978	122,592	95.79	120,834	94.42	108,108	84.47	1,287
LT	Telefon	100,950	96,330	95.42	95,362	94.46	89,802	88.96	1,081
LZ	Zapatilla	91,928	86,435	94.02	84,630	92.06	81,872	89.06	1,294

Columns indicate the number of raw input reads (Reads in), reads retained after trimming (Reads out), trimming efficiency, successfully merged reads, merging percentage, and the number, percentage of high-quality reads after filtering and the final ASV numbers.

#### 3.1.1 Comparative overview of bacterial communities in Arctic and Antarctic lakes

**Figure 2:**
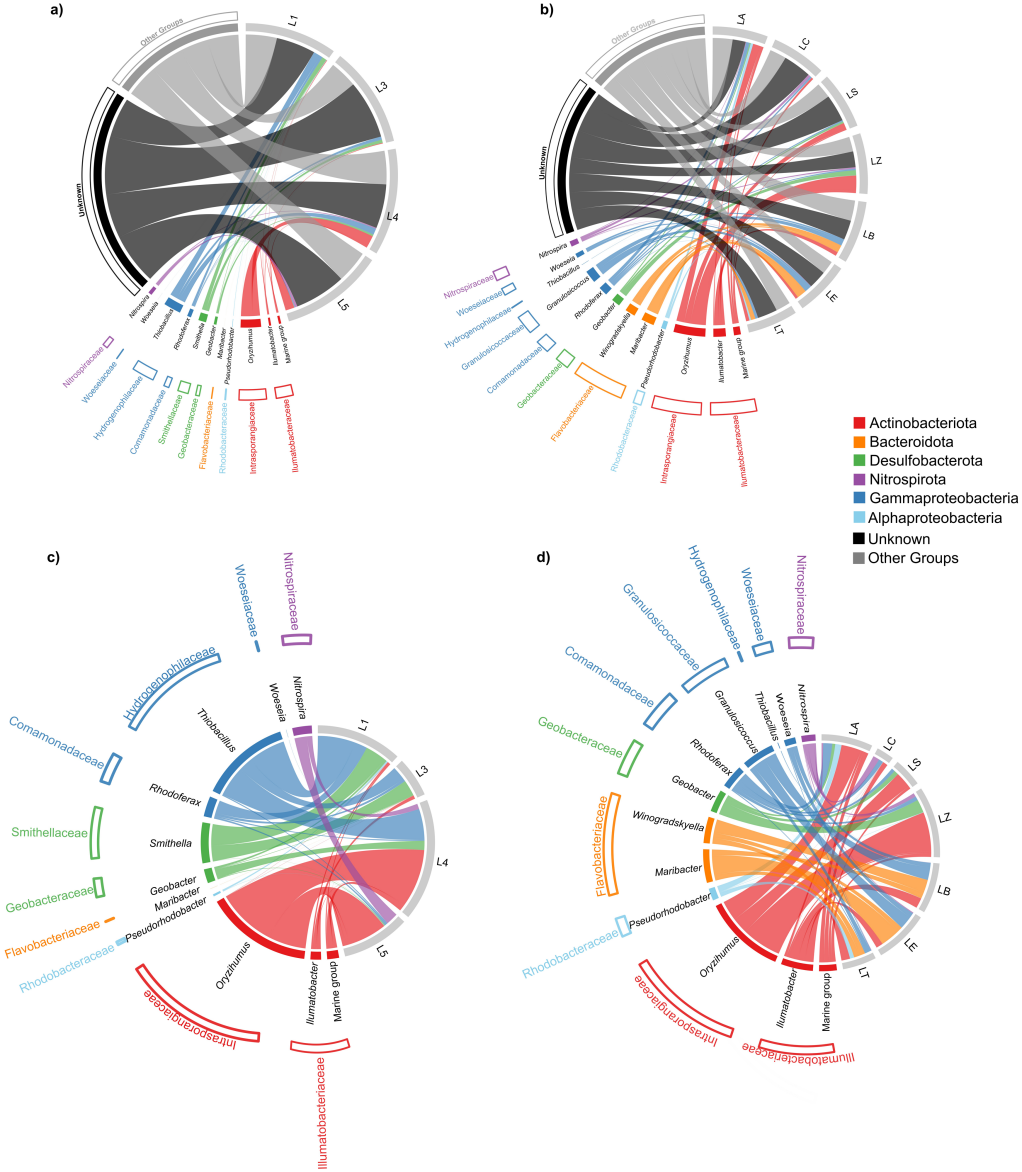
Chord diagrams depicting the taxonomic composition of sediment bacterial communities in polar lakes. Arctic lake L2 is not included in the microbial community dataset and therefore does not appear in this figure. Sector size and chord width are proportional to relative abundance and indicate the contribution of each taxon to each lake. For visualization, only higher phylogenetic groups with relative abundance >2% were displayed; taxa below this threshold were grouped as “Other groups,” while unassigned sequences are reported as “Unknown.” **(a)** Arctic lakes (L1, L3, L4, L5): links between lakes and dominant bacterial groups including Other groups and Unknown. **(b)** Antarctic lakes (LA, LC, LS, LZ, LB, LE, LT): links between lakes and dominant bacterial groups including Other groups and Unknown. **(c)** Focus on the most representative taxa in Arctic lakes, highlighting the main lake–taxon associations. **(d)** Focus on the most representative taxa in Antarctic lakes, highlighting the main lake–taxon associations. Colours correspond to the major phyla/classes reported in the legend (Actinobacteriota, Bacteroidota, Desulfobacteriota, Nitrospirota, Gammaproteobacteria, Alphaproteobacteria).

In general, at the phylum level five main phyla were present in both polar areas, namely Actinobacteriota (average 18 and 21% in Arctic and Antarctic lake sediment, respectively), Bacteroidota (average 24% in both polar areas), Alphaproteobacteria (average 7 and 19% in Arctic and Antarctic lake sediment, respectively), Gammaproteobacteria (9% in both polar areas) and partially Desulfobacterota (average 11 and 4% for Arctic and Antarctica, respectively) (Figure 2).

In Arctic Lake sediments (Figures 2a,c), Actinobacteria were most abundant in L4 (29%) and L5 (33%), while in Antarctic lakes they dominated in LS (30%), LA (31%), and LZ (43%). Bacteroidota showed high relative abundance in Arctic lakes L1 (29%) and L3 (47%), and in Antarctic lakes LS (21%), LT (36%), LB (34%), and LE (48%). Alphaproteobacteria were primarily detected in L1 and L5 (10%) in the Arctic, and in LT (41%), as well as LB and LC (both 23%) in Antarctica. Gammaproteobacteria were prominent in Arctic lakes L1 (31%), L4 (22%), and L5 (18%), and in Antarctic lakes LC (25%), L4 (24%), and LB (23%). Desulfobacterota were mainly found in Arctic lakes L3 (16%), L4 (11%), and L1 (10%), with LZ (10%) representing their main site of occurrence in Antarctica.

At the family level, few taxa were shared between the two polar regions. The most representative was *Intrasporangiaceae* (phylum: Actinobacteriota), with an average relative abundance of 5% in the Arctic and 8% in Antarctica. This family was particularly abundant in Arctic lakes L4 (13%) and L5 (7%), and in Antarctic lakes LZ (23%), LS (16%), and LA (14%). Within the phylum *Bacteroidota*, the vadinHA17 group was primarily detected in Arctic lakes, especially L3 (34%) and L1 (15%), while *Flavobacteriaceae* dominated in Antarctic lakes LE (42%), LT (33%), and LB (27%). Additionally, *Rhodobacteraceae* (phylum: Proteobacteria) were mainly observed in Antarctic lakes, particularly in LT (35%), LB (16%), and LE (10%). Most genera were detected at relative abundances exceeding 5%. Illumatobacter affiliates accounted for an average of 5% in the Antarctic brackish lakes LB, LE, and LT. In addition, the genera *Maribacter, Maritimimonas, Winogradskyella, Granulosicoccus,* and *Sulfitobacter* were also notably abundant in brackish lakes. *Maribacter* and *Maritimimonas* showed a high presence in lake LE (11 and 10%, respectively), while *Winogradskyella* was especially abundant in lake LB (8%). *Granulosicoccus* showed a high percentage in both LB and LE (8 and 7%, respectively), whereas the genus Sulfitobacter was most abundant in lake LT (9%). The genus *Geobacter* was detected in Antarctic lakes LA (2%), LS (3%), and LZ (8%), whereas in the Arctic it exceeded 1% only in L4 (1%). The genera *Arenimonas* and *Rhodoferax* were predominantly recovered in Antarctic Lake LA (6 and 5%, respectively) and were almost absent in Arctic lakes. The MND1 group, which accounted for 6% in Lake LC, was present at low abundance in the other Antarctic lakes and was absent in the Arctic. Similarly, members of *Rhodanobacter* were found in Lake LS (5% of total sequences) and were absent from all other lakes investigated. Finally, a high proportion of unknown genera was detected in both Arctic and Antarctic lake sediments (averaging 46 and 31%, respectively). Results lake by lake are reported below.

#### 3.1.2 Arctic lakes

##### 3.1.2.1 Lake Solvannet (L1)

Gammaproteobacteria and Bacteroidota were similarly predominant (31 and 29%, respectively), followed by Desulfobacterota and Alphaproteobacteria (both at 10%) (Figures 2a,c). At the family level, the predominance of Bacteroidetes vadinHA17 (14%; phylum Bacteroidota) was observed, followed by Comamonadaceae (12%) and Hydrogenophilaceae (8%), belonging to the Proteobacteria. In addition, 10% of the families detected were unknown. Finally, at the genus level, *Thiobacillus* (7%) was among the most represented genera. Most sequences were assigned to unknown genera (37%).

##### 3.1.2.2 Lake Knudsenheia (L3)

Bacteroidota was the most abundant phylum (46%), followed by Desulfobacterota (16%) and Actinobacteriota (8%). At the family level, the predominance of group Bacteroidetes vadinHA17 (34%; phylum Bacteroidota) was observed, followed by Desulfosarcinaceae (6%; phylum Campylobacterota) and Prolixibacteraceae (5%; phylum Bacteroidota). In addition, 16% of the sequence-detected families were unknown. Finally, at the genus level, *Desulfatirhabdium* (4%) was the most represented genus. Most sequences were assigned to unknown genera (55%).

##### 3.1.2.3 Lake Storvatnet (L4)

Actinobacteriota was the predominant phylum (29%), followed by Gammaproteobacteria (22%) and Desulfobacterota (11%). At the family level, the predominance of Intrasporangiaceae (13%; phylum Actinobacteriota) was observed, followed by Hydrogenophilaceae (6%; phylum Proteobacteria) and Syntrophobacteraceae (5%; phylum Desulfobacterota). In addition, 27% of detected families were unknown. At the genus level, *Oryzihumus* affiliates accounted for 11% of total sequences. Finally, most sequences were assigned to unknown genera (42%).

##### 3.1.2.4 Lake Tvillingvatnet (L5)

Actinobacteriota was the predominant phylum (33%), followed by Gammaproteobacteria (15%) and Bacteroidota and Alphaproteobacteria (12 and 10%, respectively). At the family level, Intrasporangiaceae (7%; phylum Actinobacteriota) and Bacteroidetes vadinHA17 (5%; phylum Bacteroidota) were the most abundant, also a higher value of unknown families (27%) was observed. At the genus level, the most relevant genera were *Oryzihumus* (7%) and *Gaiella* (4%). Many sequences were assigned to unknown genera (48%).

#### 3.1.3 Antarctic lakes

##### 3.1.3.1 Lake Argentina (LA)

Actinobacteriota was the most abundant phylum (31%), followed by Gammaproteobacteria (24%) and Alphaproteobacteria (14%) (Figures 2b,d). At the family level, the predominance of Intrasporangiaceae (14%; phylum Actinobacteriota) was observed, followed by Comamonadaceae (8%; phylum Proteobacteria), Rhodobacteraceae, and Sphingomonadaceae (both at 5%; both among phylum Proteobacteria). In addition, 19% of detected families were unknown. Finally, at the genus level, Oryzihumus (12%) was among the most abundant genera retrieved. Most of the sequences were assigned to unknown genera (19%), followed by.

##### 3.1.3.2 Lake Ballaneros (LB)

Bacteroidota was the most abundant phylum (34%), followed by Alphaproteobacteria and Gammaproteobacteria (both at 23%) and Actinobacteriota (12%). At the family level, the predominance of Flavobacteriaceae (27%; phylum Bacteroidota) was observed, followed by Rhodobacteraceae (16%; phylum Proteobacteria). At the genus level, the most found genera were *Winogradskyella* (8%) and *Granulosicoccus* (7%). Many sequences were assigned to unknown genera (29%).

##### 3.1.3.3 Lake Crater (LC)

Gammaproteobacteria (25%) and Alphaproteobacteria (23%) were the most abundant phyla, followed by Actinobacteriota (16%) and Bacteroidota (12%). At the family level, the predominance of Sphingomonadaceae (11%; phylum Proteobacteria) was observed, followed by Nitrosomonadaceae and Comamonadaceae (both at 7%; both among Proteobacteria). In addition, 26% of detected families were unknown. Finally, at the genus level, the MND1 group (a group of ammonia-oxidizing bacteria, belonging to the Nitrosomonadaceae family and Betaproteobacteria phylum) (6%) was retrieved as the most abundant. Many sequences were assigned to unknown genera (42%).

##### 3.1.3.4 Lake Extremadura (LE)

Bacteroidota was the most abundant phylum (48%), followed by Gammaproteobacteria (19%) and Alphaproteobacteria (15%). At the family level, the predominance of Flavobacteriaceae (42%; phylum Bacteroidota) was observed, followed by Rhodobacteraceae and Granulosicoccaceae (10 and 8%; both among phylum Proteobacteria). Finally, at the genus level the genera *Maribacter* (11%) and *Maritimimonas* (10%) were the most abundant. Many sequences were assigned to unknown genera (27%).

##### 3.1.3.5 Lake Sofia (LS)

Actinobacteria were the most abundant phylum (30%), followed by Bacteroidota (21%) and Gammaproteobacteria (18%). At the family level, the predominance of Intrasporangiaceae (16%; phylum Actinobacteriota) was observed, followed by Acetobacteraceae (9%; phylum Proteobacteria) and Comamonadaceae (7%; phylum Proteobacteria). In addition, 15% of detected families were unknown. Finally, at the genus level, the most abundant genus was Oryzihumus (9%), and in this case, many sequences were assigned to unknown genera (41%).

##### 3.1.3.6 Lake Telefon (LT)

Alphaproteobacteria were the most abundant phylum (41%), followed by Bacteroidota (36%), Gammaproteobacteria (9%), and Actinobacteriota (8%). At the family level, Rhodobacteraceae and Flavobacteriaceae were equally predominant (35 and 33%; phyla Proteobacteria and Bacteroidota, respectively), followed by Ilumatobacteraceae (5%; phylum Actinobacteriota) and Sphingomonadaceae (4%; phylum Proteobacteria). At the genus level, *Yoonia-Loktanella* and *Sulfitobacter* (both at 9%) and *Ilumatobacter* (5%) were the most retrieved genera, while many sequences were assigned to unknown genera (30%).

##### 3.1.3.7 Lake Zapatilla (LZ)

Actinobacteria were the most abundant phylum (43%), followed by Gammaproteobacteria (17%) and Desulfobacteria (10%). At the family level, the predominance of Intrasporangiaceae (23%; phylum Actinobacteriota) was observed, followed by Geobacteraceae (8%; phylum Desulfobacterota), Comamonadaceae and Gemmatimonadaceae (both at 5%; phyla Proteobacteria and Gemmatimonadota respectively). Additionally, 11% of the detected families were unknown. Finally, at the genus level, most sequences were assigned to *Oryzihumus* (23%) and *Geobacter* (8%). A significant number of sequences were also assigned to unknown genera (24%).

### 3.2 Persistent organic pollutants (POPs)

**Figure 3:**
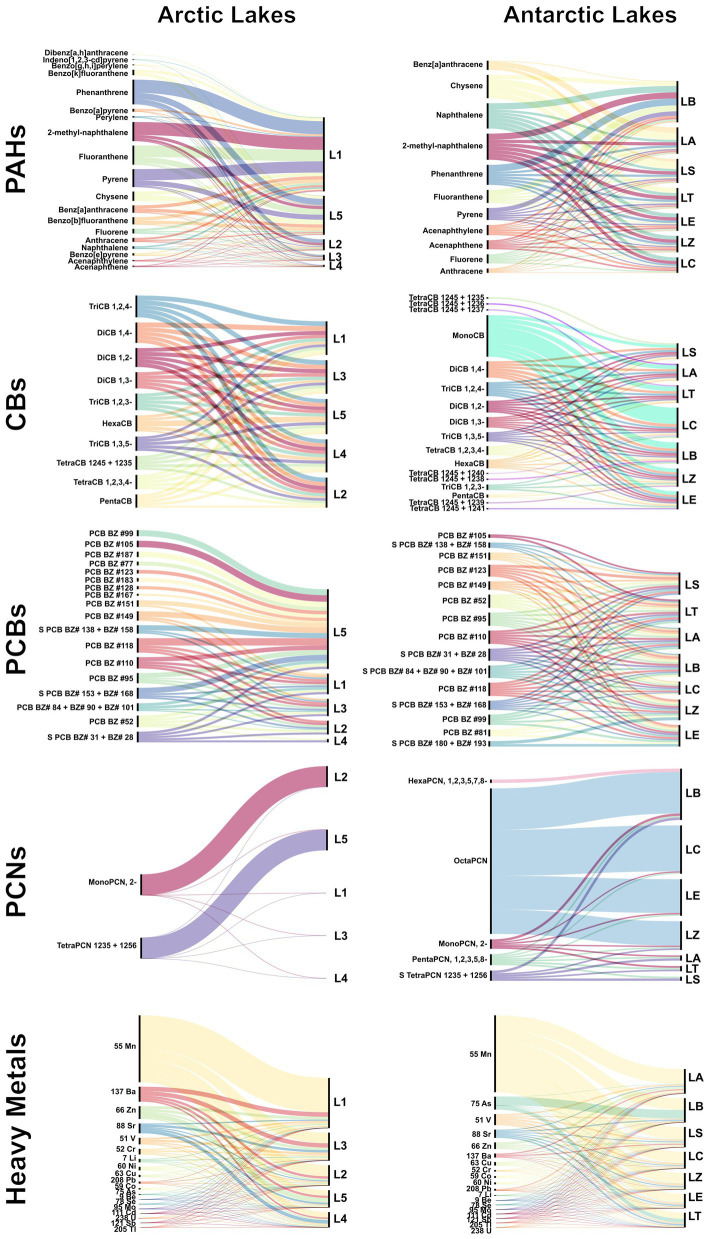
Sankey plots summarizing the distribution of the analysed organic contaminants and trace elements in lake surface sediments from the Arctic (left column) and Antarctica (right column). For each contaminant class—PAHs, chlorobenzenes (CBs), PCBs, PCNs, and heavy metals—individual compounds/congeners (or isotopes, for metals) are shown on the left and connected to the corresponding lakes on the right. The width of each flow is proportional to the relative contribution of each analyte within its class for each lake.

Overall, PAHs (∑PAH range 2.6–686.1 ppb) and CBs (∑CB range 1.8–3.5 ppb) were more abundant than PCBs (∑PCB range 0.2–1.9 ppb) and PCNs (∑PCN range 0–2.7 ppb). Across both regions, PAHs dominated the POP profiles, but at very different magnitudes: Arctic lakes showed much higher ∑PAH (18.5–686.1 ppb) than Antarctic lakes (2.6–6.3 ppb). Conversely, CBs and PCBs were higher in Antarctica (∑CB 11.8–24.0 ppb; ∑PCB 1.9–3.6 ppb) than in the Arctic (∑CB 3.1–3.5 ppb; ∑PCB 0.1–1.9 ppb). PCNs were consistently the least abundant, being ≤0.06 ppb in the Arctic and lower in Antarctica. The complete dataset of all analysed POPs is shown in Supplementary Table S2. The distribution of all organic contaminants in the analysed lakes is graphed in Figure 3 and detailed below.

#### 3.2.1 Arctic lakes

**Figure 4:**
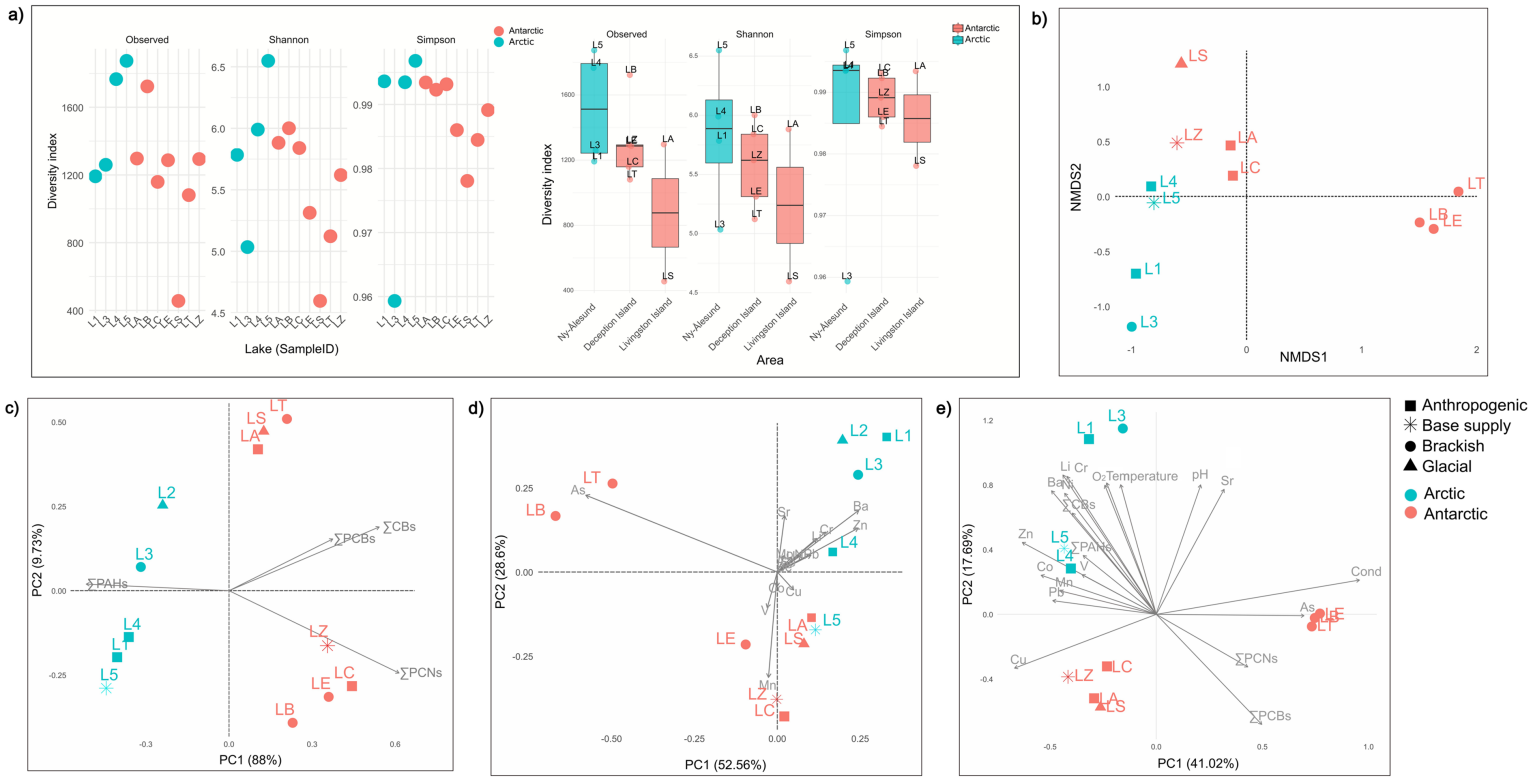
Alpha diversity and multivariate ordination analyses conducted on microbial community composition and relation with the analysed pollutants and environmental recovery factors. **(a)** Alpha diversity representation in analysed sediment samples: on the right, alpha diversity *per* lake; on the left, alpha diversity *per* analysed regions. **(b)** Non-metric multidimensional scaling (nMDS) plot based on Bray–Curtis dissimilarity of microbial community composition across lake samples. The analysis was conducted on the total set of retrieved ASVs at a stress of 0.0554. **(c)** PCA biplots showing the distribution of samples based on all organic contaminants analysed as the sum of ΣPAHs (polycyclic aromatic hydrocarbons), ΣPCBs (polychlorinated biphenyls), ΣCBs (chlorobenzenes), and ΣPCNs (polychlorinated naphthalenes) retrieved in all analysed samples. **(d)** PCA biplots showing the distribution of samples based on all trace metals analysed. **(e)** PCA biplot of lake-sediment prokaryotic communities, retrieved pollutants, and measured environmental factors. Arrows represent the environmental fit vectors of physico-chemical and contaminant variables (e.g., ΣPAHs, ΣPCBs, ΣPCNs, ΣCBs, Li, V, Cr, Mn, Co, Ni, Cu, Zn, As, Sr, Ba, Pb, Temperature, pH, O_2_, Cond). For all the panels the colours and shapes of the points represent the geographic location and sample type, respectively, as indicated in the legend. Here the analysed lakes: Solvannet (L1), Glacier (L2), Knudsenheia (L3), Storvatnet (L4), Tvillingvatnet (L5), Argentina (LA), Sofia (LS) Ballaneros (LB), Crater (LC), Extremadura (LE), Telefon (LT), and Zapatilla (LZ). Lake Glacier (L2) is included in the chemical ordinations (panels **b,c**) but excluded from microbial-based ordinations (panels **a,b,e**) due to sample loss.

Overall, PAHs were the most abundant group of contaminants (∑PAH range: 18.5–686.1 ppb), followed by CBs (∑CB range: 3.1–3.5 ppb), with PCBs (∑PCB range: 0.1–1.9 ppb) and PCNs being the least concentrated (∑PCN range: 0–0.06 ppb). POPs detected in lake sediment samples are listed in Supplementary Table S2a and graphed in Figure 4. Among the PAHs, phenanthrene was the most abundant compound across all lakes, with mean concentrations ranging from 5.4 ppb in Lake Glacier to 123.8 ppb in Lake Tvillingvatnet. It was followed by 2-methyl-naphthalene, fluoranthene, and pyrene, which showed the highest value, all in Lake Tvillingvatnet (122.5, 108.4, and 106.3 ppb, respectively). Lake Solvannet also showed high concentrations of the same PAHs; phenanthrene was measured at 58.3 ppb, while 2-methyl-naphthalene, fluoranthene, and pyrene had the respective values of 28.4, 52.9, and 47.4 ppb. Among chlorobenzenes (CBs), monochlorobiphenyls (mono-CBs) were the predominant group in all lakes, with average concentrations ranging from 0.43 ppb in Lake Glacier to 0.76 ppb in Lake Storvatnet (Supplementary Table S2a).

#### 3.2.2 Antarctic lakes

Overall, PAHs were the most abundant group of contaminants (∑PAH range: 2.6–6.3 ppb), followed by CBs (∑CB range: 11.8–24.0 ppb) and PCBs (∑PCB range: 1.9–3.6 ppb), with PCBs being the least concentrated (∑PCB range: 0.7–1.03 ppb). POPs detected in lake sediment samples are listed in Supplementary Table S2b and graphed in Figure 3. Among the PAHs, 2-methyl-naphthalene was the most abundant, with mean concentrations ranging from 0.5 ppb to 1.02 ppb, respectively, in Lake Crater and Lake Ballaneros. Regarding CBs, mono-CBs were the most prevalent in all lakes, with concentrations ranging from 0.4 ppb in Lake Sofia to 1.9 ppb in Lake Crater. Overall, PCNs concentrations were relatively low, but tetraPCN (particularly ∑ tetraPCN1,2,3,5- + 1,2,5,6-) showed some prevalence over other congeners in Lakes Argentina and Sofia. For PCBs, concentrations were generally low, with no levels exceeding 0.5 ppb (∑ of hexachlorobiphenyl) in Lake Sofia. However, in certain lakes such as Lake Ballaneros, Lake Telefon, and Lake Zapatilla, pentachlorobiphenyls were also detected above the relevant concentration (Supplementary Table S2b).

### 3.3 Concentration of trace metals

Overall, trace elements levels varied widely. Manganese (Mn) showed the highest mean concentration (272 ppm), followed by zinc (Zn, mean 36 ppm), barium (Ba, mean 36 ppm), and strontium (Sr, mean 34 ppm). Thallium (Tl) showed the lowest mean concentrations (0.10 ppm). Data on trace element concentrations are detailed in Supplementary Table S2c and in Figure 3.

#### 3.3.1 Arctic lakes

Lakes Tvillingvatnet (L5), Knudsenheia (L3), and Solvannet (L1) showed particularly high enrichment in trace metals (ppm), with notably elevated manganese (Mn; 236–760), zinc (Zn; 56–102), vanadium (V; 39–51), copper (Cu; 10–13), and nickel (Ni; 22–24). In contrast, Storvatnet (L4) and Glacier (L2) generally exhibited lower concentrations for most analytes; a notable exception was strontium (Sr) in Lake Glacier (L2), which reached 75 ppm (Supplementary Table S2c and Figure 3).

#### 3.3.2 Antarctic lakes

Lakes Argentina (LA), Sofia (LS), and Ballaneros (LB) showed comparatively elevated trace-metal levels, with particularly high manganese (Mn; 275–349 ppm) and, to a lesser extent, zinc (Zn; 9–56 ppm), copper (Cu; 4–19 ppm), lead (Pb; 2–24 ppm), and lithium (Li; 1.04–5 ppm). Arsenic (As) displayed a marked peak in Lake Ballaneros (LB) (196 ppm). In contrast, Lakes Extremadura (LE), Telefon (LT), Crater (LC), and Zapatilla (LZ) generally exhibited lower concentrations for many analytes (e.g., Li ≤ 3 ppm, Cu ≤ 14 ppm, Zn ≤ 18 ppm, Pb ≤ 1 ppm). However, Mn remained comparatively high in this group (142–267 ppm). A notable exception was vanadium (V) in LZ Anta, which reached 47 ppm (Supplementary Table S2c and Figure 3).

### 3.4 Statistical analyses

Bacterial α-diversity varied among lakes but showed overlapping ranges between regions and categories (Figure 4a). Observed richness spanned from ~400 to >1,600 ASVs per sample, with the lowest values in the Antarctic glacial lake LS (456) and the highest in the Arctic lake L5 (1,874). Shannon and Simpson indices were ranged between 4.5–6.5 and 0.96–0.99, respectively, and boxplots by area indicated substantial within-area variability, with both Arctic and Antarctic lakes contributing to the full diversity spectrum. A PERMANOVA using the model bray ~ Regions (Arctic and Antarctica) + Areas (Ny-Ålesund, Deception Island, Livingston Island) (9,999 permutations) showed that the this combination explained 45.4% of the variance in community dissimilarity (Model: df = 3, Sum of Sqs = 1.8895, *R*^2^ = 0.45436, *F* = 1.6654, *p* = 8.0 × 10^−4^), with the remaining 54.6% attributed to residual variation. Non-metric multidimensional scaling of Bray–Curtis dissimilarities calculated using the complete ASVs table (Figure 4b) was performed with an analysis stress of 0.0554, indicating a reliable ordination. The analysis revealed a clear separation of the brackish Antarctic lakes (LT, LB, and LE), which formed a distinct cluster apart from all other lakes. The remaining Antarctic lakes were also grouped, but they occupied a different region of the ordination space in the upper-left quadrant. The Arctic samples appear on the left, forming a group with less overlap (L4 and L5) with the Antarctic assemblages (LA, LC, LS, and LZ). Overall, the nMDS ordination highlights a clear biogeographic structuring of bacterial communities, with lakes clustering primarily by region and secondarily by lake category. PCA (Figure 4c) evidenced the primary drivers of POPs contaminant profiles across the studied polar lacustrine environments. The PC1 axis accounts for 88.0% of the variance, and a secondary axis accounts for 9.73%. Along the main gradient, all Arctic lakes had negative scores and were grouped on the side associated with ΣPAHs. In contrast, all Antarctic lakes had positive scores and clustered towards the chlorinated POPs, with ΣCBs, ΣPCBs, and ΣPCNs showing strong, nearly collinear loadings. Within this Antarctic cluster, two separated groups were shown in the analysis. Lakes LB, LE, LC, and LZ were grouped along the ΣPCNs vector. Otherwise, the lakes LS, LA, and LT collectively shifted upward along the second axis, indicating a POP mixture distinct from that of the other lakes. For trace metals, PCA (Figure 4d) revealed a clear horizontal gradient separating Arctic and Antarctic lakes. Arctic Lakes L1, L2, and L3 clustered on the right-hand side of the plot, in the direction of Ba, Zn, and Sr, indicating relatively higher concentrations of these elements; the Arctic lake L4 occupied an intermediate position, still on the same side of the gradient but closer to the origin. In contrast, Antarctic Lakes LT LB were grouped on the left, aligned with the As vector and plotted at high values on the vertical axis, whereas the Antarctic Lakes LC, LE, and LZ lay lower and closer to Mn. Finally, Antarctic Lakes LA and LS, and Arctic Lake L5, were grouped in the bottom-right portion of the graph, indicating association with the Cu vector. A combined PCA was performed, including all retrieved results, both chemical and biological, and environmental parameters reported by Rappazzo et al. (2025a), to understand their potential relationships across the analysed lakes (Figure 4e). PC1 accounted for 41.02% and PC2 for 17.69% of the variance. Conductivity, ΣPCBs, and ΣPCNs loaded strongly and positively on PC1, whereas pH, water temperature, and several lithogenic elements (e.g., Li, Cr, Sr) contributed primarily to PC2. Arctic lakes L1–L3 plotted in the upper-left quadrant, in the direction of higher pH, temperature, and conservative elements but away from the conductivity/POP vectors, whereas L4 and L5 were shifted towards the center and slightly towards the POP and metal vectors (including Zn). Among Antarctic Lakes, LB, LT, and LE clustered in the lower-right region of the plot, aligned with conductivity and chlorinated POPs. In contrast, LC, LA, LS, and LZ were grouped in the lower-left region, closer to Cu and Mn. This combined PCA revealed an association between microbial composition and contaminants and underscored the influence of physicochemical parameters. In-depth correlation analyses were conducted among the 20 most abundant bacterial families in each region (excluding unclassified families). The complete set of environmental and contaminant variables (Supplementary Figure S1) indicated that, in the Arctic subset, Spearman coefficients for temperature, pH, conductivity, POP sums, and trace metals ranged from negative to positive. However, none were significant at *p* ≤ 0.05. In contrast, in the Antarctic subset, several dominant families exhibited significant correlations (*p* ≤ 0.05). Among the physical–chemical parameters, only conductivity showed significant positive correlations (*p* ≤ 0.05) with Rhodobacteraceae, Granulosicoccaceae (Proteobacteria), and Flavobacteriaceae, and negative correlations (*p* ≤ 0.05) with Acetobacteraceae (Proteobacteria) and Chitinophagaceae (Bacteroidota). In the specific POP groups, only PCBs showed a negative correlation (*p* ≤ 0.05) with Rhizobiaceae (Proteobacteria) and Nitrospiraceae (Nitrospirota). Metals and trace elements showed numerous significant correlations with selected bacterial families, particularly with nonessential metals such as Be, Cd, and U, which showed positive correlations (*p* ≤ 0.001, *p* ≤ 0.05, and *p* ≤ 0.05, respectively) with the Microbacteriaceae (Actinobacteriota) family.

## 4 Discussion

Polar lakes are increasingly recognized as sentinels of environmental change and natural laboratories for testing biotic responses to multiple stressors in rapidly warming high-latitude regions (Picazo et al., 2021; Calizza et al., 2022; Saros et al., 2022). In this context, the comparison between Arctic lakes in Svalbard and Antarctic lakes in the South Shetland Islands/Deception Island highlights two clearly distinct contaminant signatures that reflect differences in emission sources, transport pathways, and depositional regimes.

### 4.1 Ecological significance of contrasting contaminant profiles

Arctic sediments were dominated by PAHs, in line with their origin as combustion-derived pollutants transported over long distances and strongly partitioning into particles and sediments, but also with additional inputs from local and historical activities, including past coal mining and associated traffic near Ny-Ålesund (Lehmann-Konera et al., 2020; Millar et al., 2021; Meierdierks et al., 2022; Vecchiato et al., 2023). In contrast, Antarctic sediments showed relatively higher levels of CBs and PCBs, a combination that is consistent with their role as globally transported POPs emitted at mid-latitudes, coupled with more local and regional sources linked to research stations, former whaling activities, and coastal infrastructure (Cabrerizo et al., 2012; Kallenborn et al., 2013; Combi et al., 2017; Zhang et al., 2013; Reed et al., 2018; Casal et al., 2019; Sutilli et al., 2019; Deng et al., 2020; Nash et al., 2021). Trace metals added an additional environmental gradient, with manganese consistently emerging as the dominant metal in both regions and with local enrichments of arsenic and lead suggesting the interplay of litho-geochemical controls, redox processes, and possible anthropogenic contributions (Lyons et al., 1985; Mentasti et al., 2003; Nędzarek et al., 2014; Shevchenko et al., 2017; Kаkareka et al., 2019; Lecomte et al., 2019). Overall, the combined analysis of PAHs, PCBs, PCNs, and CBs provides a diagnostic contaminant framework that clearly distinguishes Arctic lakes—primarily influenced by combustion-related pollutants—from Antarctic lakes, where chlorinated compounds, including under-monitored PCNs, reveal the superposition of hemispheric transport with more proximal sources (Zhang et al., 2024; Simon et al., 2023; Vorkamp et al., 2022; Muir et al., 2025).

### 4.2 Bacterial community composition

In both regions, sedimentary prokaryotic communities were dominated by a recurring set of phyla—Actinobacteriota, Bacteroidota, Alpha- and Gammaproteobacteria, and Desulfobacterota—in agreement with observations from other polar lakes and coastal systems where these groups underpin key carbon, sulfur, and metal cycles (Quero et al., 2015; Wang et al., 2016; Vázquez et al., 2017; Picazo et al., 2019; Li et al., 2020; Panwar et al., 2020; Millar et al., 2021; Tytgat et al., 2023; Wood et al., 2025). However, beneath this apparent phylum-level convergence, pronounced biogeographic patterns emerged at family and genus level, reflecting the combined influence of geological history, physicochemical conditions, marine connectivity, and contaminant loads specific to each lake.

A critical outcome is the high fraction of unclassified genera, especially in Arctic sediments, where unknown genera accounted for a substantial portion of the community and were dominant in several lakes. This pattern highlights both a strong potential for phylogenetic novelty in polar sediments and the limitations of current taxonomic databases in capturing microbial diversity in high-latitude environments (Pessi et al., 2023; Tytgat et al., 2023). Among classified taxa, Intrasporangiaceae (Actinobacteriota), particularly *Oryzihumus*, emerged as one of the most ubiquitous and abundant lineages across both regions, being especially prominent in Arctic Lake Storvatnet and Lake Tvillingvatnet and in Antarctic Lake Zapatilla, Lake Sofia, and Lake Argentina. Their distribution suggests shared niches characterized by oligotrophy, stress resistance, potential metal tolerance, and the ability to utilize complex substrates under cold conditions (Fernández-López et al., 2024).

In Arctic lakes, the enrichment of Desulfobacterota, together with families such as Bacteroidetes vadinHA17 (Bacteroidota), Desulfosarcinaceae (Campylobacterota/Desulfobacterota-affiliated), Syntrophobacteraceae (Desulfobacterota), and Hydrogenophilaceae and Comamonadaceae (Proteobacteria), is consistent with the presence of reduced microenvironments in surface sediments where sulfate reduction, syntrophic metabolisms, and metal mobilization may be tightly coupled (Hawley et al., 2017; Vigneron et al., 2021; Magnuson et al., 2023). At the genus level, *Desulfatirhabdium* in Lake Knudsenheia and *Thiobacillus* in Lake Solvannet illustrate the coexistence of sulfur-oxidizing and sulfur-reducing guilds, while *Oryzihumus* and *Gaiella* in Lake Tvillingvatnet and *Oryzihumus* affiliates in Lake Storvatnet point to versatile Actinobacteriota adapted to oligotrophic and metal-impacted niches.

In Antarctic lakes, and especially in brackish systems from Deception Island, communities carried a strong marine imprint. Flavobacteriaceae and Rhodobacteraceae were particularly abundant in lakes Ballaneros, Extremadura, and Telefon, together with Granulosicoccaceae, Sphingomonadaceae, Comamonadaceae, Acetobacteraceae, Ilumatobacteraceae, and Geobacteraceae. At genus level, *Winogradskyella* and *Granulosicoccus* in Lake Ballaneros, *Maribacter* and *Maritimimonas* in Lake Extremadura, *Sulfitobacter* and *Yoonia–Loktanella* in Lake Telefon, and *Ilumatobacter* affiliates in brackish lakes collectively reflect typical coastal and brackish assemblages associated with algal-derived organic matter degradation, salinity gradients, and high productivity (Abell and Bowman, 2004; Quiroga et al., 2024; Wang et al., 2020; Picazo et al., 2021). The consistent presence of *Ilumatobacter* (Actinobacteriota) in brackish lakes, together with Comamonadaceae and Acetobacteraceae, further indicates flexible strategies for coping with variable redox, organic matter quality, and contaminant exposure.

The occurrence of *Geobacter* in multiple Antarctic lakes, and its strong contribution in Lake Zapatilla, points to potential metal-respiring metabolisms and redox control over elements such as iron, manganese, and vanadium, in agreement with the local metal profiles and the presence of stratified sediments enriched in transition metal oxides (Beam et al., 2020; Ru et al., 2019; Odisi et al., 2023). Additional genera such as *Arenimonas* and *Rhodoferax* (notably in Lake Argentina), the MND1 group of ammonia-oxidizing Betaproteobacteria in Lake Crater, and members of *Rhodanobacter* in Lake Sofia underline further functional specialization along nitrogen and redox gradients. The coexistence, within the same archipelago, of lakes with a strong marine–coastal signature (e.g., Ballaneros, Extremadura, Telefon) and others with weaker marine influence (e.g., Argentina, Sofia, Zapatilla, Crater) underscores how relatively small geomorphological and hydrological differences can translate into markedly distinct microbial assemblages at short spatial scales.

### 4.3 Diversity patterns and community–environment relationships

Alpha-diversity estimates indicated that sediment bacterial richness and evenness were of the same order of magnitude in Arctic and Antarctic lakes, with no statistically significant differences between regions or among sites. Nevertheless, individual lakes spanned a wide diversity gradient: Tvillingvatnet and Ballaneros exhibited the highest richness values, whereas some Antarctic glacial and brackish systems displayed comparatively lower diversity. This firm within-lake heterogeneity, superimposed on similar regional medians, indicates that local environmental conditions and disturbance histories exert greater control on alpha diversity than broad geographic location alone (Pearman et al., 2022). Multivariate analyses of *β*-diversity indicated that both region (Arctic vs. Antarctic) and lake sites significantly contributed to community structuring. At the same time, dispersion tests supported that these differences were not primarily driven by unequal within-group dispersion (Oksanen et al., 2022). The nMDS ordination clearly separated brackish Antarctic lakes from freshwater systems, with non-brackish Antarctic lakes occupying an intermediate position and Arctic lakes forming a distinct cluster in ordination space. This pattern is consistent with previous studies identifying salinity, marine connectivity, and trophic state as primary drivers of microbial biogeography in polar lakes (Quero et al., 2015; Picazo et al., 2019; Wang et al., 2020; Picazo et al., 2021). PCAs based on POPs and metals revealed that the separation between Arctic and Antarctic lakes is mirrored by well-defined chemical gradients: Arctic lakes were more strongly associated with PAHs and specific lithogenic elements, whereas Antarctic lakes, particularly those with higher conductivity, were more strongly associated with CBs, PCBs, PCNs, and selected metals. The combined PCA, which integrated chemical data, physicochemical parameters, and diversity metrics, indicated that community composition followed gradients in which conductivity, chlorinated POPs, and certain non-essential metals were particularly influential in Antarctic lakes. At the same time, pH, water temperature, and lithogenic elements played a stronger role in Arctic systems (Rappazzo et al., 2025a). These findings are consistent with the role of sediments as integrative archives of environmental pressures, simultaneously recording contaminant inputs and the responses of the microbial assemblages that transform or sequester them (Chiaia-Hernández et al., 2022; Chen et al., 2019; Lyautey et al., 2021). Correlation analyses focused on the most abundant families further supported region-specific linkages between community composition and environmental drivers. In the Antarctic subset, significant associations emerged between conductivity and marine/coastal families such as Rhodobacteraceae, Granulosicoccaceae, and Flavobacteriaceae, as well as negative associations with Acetobacteraceae and Chitinophagaceae, which may reflect differences in substrate preferences or salinity tolerance. Non-essential metals such as Be, Cd, and U showed positive relationships with Microbacteriaceae, suggesting possible adaptations or tolerance mechanisms to metal exposure (Mallick et al., 2021). In the Arctic subset, by contrast, correlations with individual environmental variables were not significant, indicating that a more integrated set of drivers may modulate community variability, or that, given the number of lakes, relationships between specific taxa and single variables remain statistically difficult to detect. The patterns observed in natural communities are broadly consistent with independent enrichment experiments performed on the same sediments, which identified bacteria capable of tolerating or transforming PCBs and withstanding metal mixtures, often enriched in Pseudomonas and members of Actinomicetota and Bacillota (Rappazzo et al., 2025a; Rappazzo et al., 2025b). Although amplicon-based approaches do not allow direct assignment of specific metabolic functions, the co-occurrence of taxa with contaminant and metal gradients, together with enrichment data, suggests that at least part of the sediment communities may actively contribute to contaminant degradation, transformation, or immobilization (Custodio et al., 2022; Zhang et al., 2025).

## 5 Conclusion

Taking together, our results show that the investigated polar lakes host highly structured bacterial communities responding to the combined influence of geography, trophic state, marine connectivity, and contaminant profiles, with clearly distinct Arctic and Antarctic chemical signatures mirrored by equally distinct microbial assemblages. While a limited set of dominant phyla is shared across regions, marked divergence at family and genus levels, coupled with the substantial fraction of unclassified genera—especially in the Arctic—highlights both strong biogeographic structuring and the extent of yet undescribed microbial diversity in polar sediments. Multivariate analyses support the view that, particularly in Antarctic lakes, persistent organic pollutants and nonessential metals, in concert with conductivity and pH, contribute significantly to community structuring, thereby confirming polar lakes as early-warning sensors of multiple interacting pressures. This Arctic–Antarctic comparative baseline provides a valuable reference for long-term monitoring of climate-driven remobilization of legacy contaminants from thawing permafrost and retreating glaciers and for assessing future changes in polar aquatic ecosystems. Moving forward, it will be essential to expand spatial and temporal coverage, couple amplicon data with multi-omic approaches, and include viral and eukaryotic components to capture responses at the level of entire ecological networks. In this perspective, polar lakes emerge not only as archives of past pressures but also as key platforms for understanding resilience, thresholds, and potential tipping points of high-latitude ecosystems under the combined influence of climate change and contamination.

## Data Availability

The datasets presented in this study can be found in online repositories. The names of the repository/repositories and accession number(s) can be found in the article/Supplementary material.

## Supplementary material

SUPPLEMENTARY FIGURE S1 Spearman correlation heatmap between the 20 most abundant bacterial families (rows; grouped by phylum) and environmental and contaminant variables (columns) in Arctic (top panel) and Antarctic (bottom panel) lake sediments. Environmental variables include water temperature, pH, dissolved oxygen and conductivity, followed by POPs (ΣCBs, ΣPAHs, ΣPCNs, ΣPCBs) and trace elements (Li–U). Colours indicate the strength and direction of the correlation (Spearman’s *ρ*), from negative (blue) to positive (red). Asterisks mark statistically significant correlations after FDR correction (^*^*p* ≤ 0.05, ^**^*p* ≤ 0.01, and ^***^*p* ≤ 0.001).
