# Supplementary figures and images for: Diverging contaminant profiles and prokaryotic assemblages in Arctic and Antarctic lake sediments
Source: Front Microbiol. 2026 Jan 21;16:1722478. doi: 10.3389/fmicb.2025.1722478 (PMC12869993; doi:10.3389/fmicb.2025.1722478)

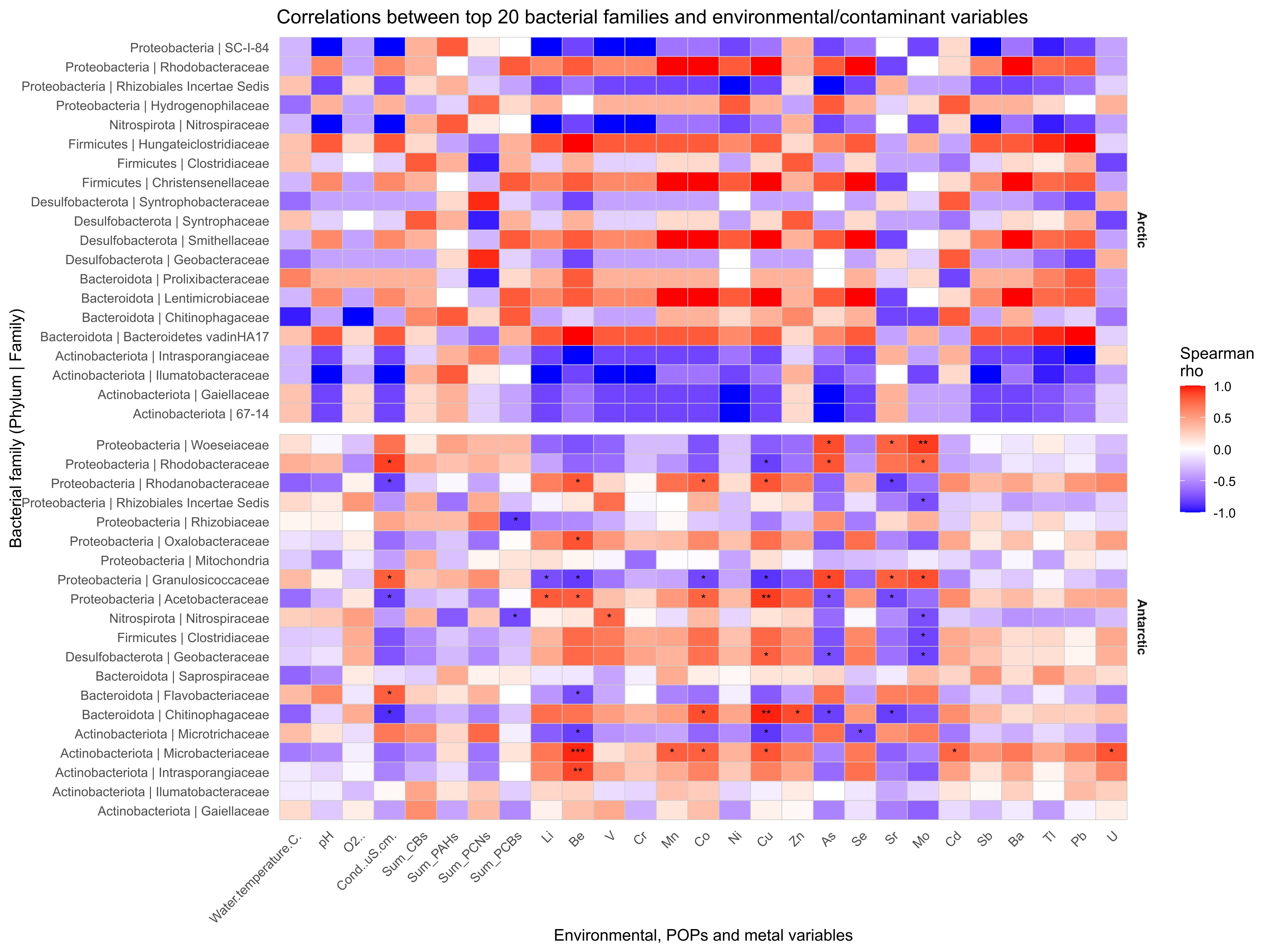

Supplement: Supplementary file 1 [file Image_1.jpeg]
