# Supplementary material for: Diverging contaminant profiles and prokaryotic assemblages in Arctic and Antarctic lake sediments
Source: Front Microbiol. 2026 Jan 21;16:1722478. doi: 10.3389/fmicb.2025.1722478 (PMC12869993; doi:10.3389/fmicb.2025.1722478)
Supplement: Supplementary file 3 [file Table_2.docx]

Tab. Suppl. 2 Distribution of the detected persistent organic pollutants such as PCBs, PCNs, CBs, PAHs, and trace metals present including non-quantifiable (n.q.) or undetermined (n.d.) compounds in the analyzed sediment samples. a) Sediments from Arctic lakes. b) Sediments from Antarctic lakes. c) Trace metal elements in the Arctic and Antarctic sediments lakes.

a)

| **Compounds** | **Lake Tvillingvatnet** | **Lake Storvatnet** | **Lake Knudsenheia** | **Lake Glacier** | **Lake Solvannet** |
| --- | --- | --- | --- | --- | --- |
| *Polycyclic Aromatic Hydrocarbons (PAHs)* |  |  |  |  |  |
| Naphthalene | 14.67 ± 0.39 | 2.49 ± 0.10 | 1.39 ± 0.08 | 0.95 ± 0.04 | 3.69 ± 0.59 |
| 2-methyl-naphthalene | 122.30 ± 4.77 | 20.93 ± 2.30 | 5.73 ± 0.59 | 2.04 ± 0.26 | 28.39 ± 5.34 |
| Acenaphthylene | 6.74 ± 0.54 | 1.32 ± 0.12 | 0.56 ± 0.02 | 0.41 ± 0.02 | 1.65 ± 0.18 |
| Acenaphthene | 6.37 ± 0.81 | 1.63 ± 0.16 | 0.75 ± 0.05 | 0.44 ± 0.01 | 2.33 ± 0.26 |
| Fluorene | 21.87 ± 1.22 | 8.53 ± 1.20 | 2.33 ± 0.40 | 0.78 ± 0.06 | 9.64 ± 0.63 |
| Phenanthrene | 123.83 ± 15.37 | 33.07 ± 5.50 | 12.67 ± 1.73 | 5.38 ± 0.63 | 58.23 ± 3.61 |
| Anthracene | 18.75 ± 1.84 | 4.21 ± 0.63 | 2.04 ± 0.42 | 0.58 ± 0.03 | 11.09 ± 1.48 |
| Fluoranthene | 108.23 ± 4.57 | 8.53 ± 0.98 | 8.62 ± 3.78 | 1.55 ± 0.13 | 53.04 ± 7.41 |
| Pyrene | 106.17 ± 5.03 | 7.38 ± 0.93 | 6.10 ± 2.58 | 2.22 ± 0.17 | 47.47 ± 3.28 |
| Benz[a]anthracene | 30.88 ± 4.18 | 3.96 ± 0.40 | 2.85 ± 1.35 | 0.85 ± 0.03 | 30.47 ± 5.23 |
| Chysene | 33.85 ± 4.25 | 4.92 ± 0.60 | 3.63 ± 1.33 | 2.09 ± 0.22 | 43.61 ± 4.35 |
| Benzo[b]fluoranthene | 32.19 ± 7.00 | 1.60 ± 0.25 | 1.81 ± 0.84 | 0.74 ± 0.06 | 31.67 ± 9.84 |
| Benzo[k]fluoranthene | 23.25 ± 8.85 | 0.78 ± 0.08 | 1.23 ± 0.59 | n.r. | 25.98 ± 11.04 |
| Benzo[e]pyrene | 12.00 ± 1.59 | 0.82 ± 0.10 | 0.96 ± 0.38 | 0.52 ± 0.11 | 7.69 ± 0.44 |
| **Compounds** | **Lake Tvillingvatnet** | **Lake Storvatnet** | **Lake Knudsenheia** | **Lake Glacier** | **Lake Solvannet** |
| Benzo[a]pyrene | 17.20 ± 2.62 | 0.93 ± 0.11 | 1.05 ± 0.18 | n.r. | 6.79 ± 0.77 |
| Perylene | 6.93 ± 0.69 | 2.39 ± 0.32 | 1.37 ± 0.29 | n.r. | 3.29 ± 0.77 |
| Indeno[1,2,3-cd]pyrene | 5.09 ± 1.42 | n.r. | n.r. | n.r. | 1.19 ± 0.33 |
| Dibenz[a,h]anthracene | 1.48 ± 0.34 | n.r. | n.r. | n.r. | n.r. |
| Benzo[g,h,i]perylene | 6.05 ± 1.82 | n.r. | 0.37 ± 0.05 | n.r. | 1.16 ± 0.38 |
|  |  |  |  |  |  |
| *Chlorobenzenes (CBs)* |  |  |  |  |  |
| MonoCB | 0.74 ± 0.01 | 0.76 ± 0.02 | 0.64 ± 0.03 | 0.43 ± 0.02 | 0.63 ± 0.08 |
| DiCB 1,3- | 0.26 ± 0.01 | 0.26 ± 0.01 | 0.28 ± 0.01 | 0.26 ± 0.00 | 0.25 ± 0.00 |
| DiCB 1,4- | 0.40 ± 0.01 | 0.31 ± 0.02 | 0.34 ± 0.01 | 0.29 ± 0.00 | 0.31 ± 0.01 |
| DiCB 1,2- | 0.31 ± 0.02 | 0.32 ± 0.02 | 0.33 ± 0.00 | 0.30 ± 0.01 | 0.27 ± 0.01 |
| TriCB 1,3,5- | 0.21 ± 0.01 | 0.21 ± 0.01 | 0.23 ± 0.01 | 0.25 ± 0.01 | 0.25 ± 0.01 |
| TriCB 1,2,4- | 0.38 ± 0.01 | 0.37 ± 0.04 | 0.38 ± 0.02 | 0.32 ± 0.01 | 0.31 ± 0.00 |
| TriCB 1,2,3- | 0.27 ± 0.01 | 0.26 ± 0.01 | 0.27 ± 0.00 | 0.27 ± 0.01 | 0.27 ± 0.01 |
| TetraCB 1245 + 1235 | 0.21 ± 0.01 | 0.20 ± 0.01 | 0.23 ± 0.01 | 0.24 ± 0.03 | 0.23 ± 0.04 |
| TetraCB 1,2,3,4- | 0.22 ± 0.01 | 0.20 ± 0.01 | 0.23 ± 0.01 | 0.26 ± 0.01 | 0.25 ± 0.01 |
| PentaCB | 0.22 ± 0.01 | 0.16 ± 0.01 | 0.23 ± 0.01 | 0.21 ± 0.06 | 0.25 ± 0.03 |
| HexaCB | 0.24 ± 0.01 | 0.14 ± 0.02 | 0.20 ± 0.02 | 0.27 ± 0.01 | 0.45 ± 0.03 |
|  |  |  |  |  |  |
| *Polychlorobiphenyls (PCBs)* |  |  |  |  |  |
| S PCB BZ# 31 + BZ# 28 | 0.10 ± 0.00 | 0.09 ± 0.00 | 0.09 ± 0.00 | 0.09 ± 0.00 | 0.11 ± 0.00 |
| PCB BZ #52 | 0.10 ± 0.01 | 0.11 ± 0.00 | 0.10 ± 0.01 | 0.06 ± 0.02 | 0.15 ± 0.01 |
| **Compounds** | **Lake Tvillingvatnet** | **Lake Storvatnet** | **Lake Knudsenheia** | **Lake Glacier** | **Lake Solvannet** |
| PCB BZ #95 | 0.05 ± 0.03 | 0.10 ± 0.02 | 0.09 ± 0.03 | n.r. | 0.22 ± 0.02 |
| S PCB BZ# 84 + BZ# 90 + BZ# 101 | 0.12 ± 0.01 | 0.04 ± 0.01 | 0.06 ± 0.01 | n.r. | 0.13 ± 0.01 |
| PCB BZ #99 | n.r. | n.r. | n.r. | n.r. | 0.27 ± 0.03 |
| PCB BZ #110 | 0.11 ± 0.00 | 0.10 ± 0.01 | 0.10 ± 0.00 | n.q. | 0.20 ± 0.03 |
| PCB BZ #77 | n.r. | n.r. | n.r. | n.r. | 0.17 ± 0.01 |
| PCB BZ #151 | 0.09 ± 0.00 | n.r. | n.r. | n.r. | 0.20 ± 0.02 |
| PCB BZ #149 | 0.09 ± 0.00 | n.r. | 0.08 ± 0.00 | n.r. | 0.25 ± 0.04 |
| PCB BZ #123 | n.r. | n.r. | n.r. | n.r. | 0.16 ± 0.01 |
| PCB BZ #118 | 0.11 ± 0.01 | 0.10 ± 0.00 | 0.11 ± 0.00 | n.r. | 0.33 ± 0.06 |
| S PCB BZ# 153 + BZ# 168 | 0.08 ± 0.00 | 0.08 ± 0.00 | 0.08 ± 0.00 | n.r. | 0.25 ± 0.04 |
| PCB BZ #105 | n.r. | n.r. | n.r. | n.r. | 0.29 ± 0.05 |
| S PCB BZ# 138 + BZ# 158 | 0.08 ± 0.01 | n.r. | 0.07 ± 0.00 | n.r. | 0.23 ± 0.04 |
| PCB BZ #187 | n.r. | n.r. | n.r. | n.r. | 0.24 ± 0.04 |
| PCB BZ #183 | n.r. | n.r. | n.r. | n.r. | 0.16 ± 0.02 |
| PCB BZ #128 | n.r. | n.r. | n.r. | n.r. | 0.14 ± 0.03 |
| PCB BZ #167 | n.r. | n.r. | n.r. | n.r. | 0.09 ± 0.01 |
| PCB BZ #177 | n.q. | n.r. | n.r. | n.r. | 0.14 ± 0.01 |
| S PCB BZ# 180 + BZ# 193 | 0.08 ± 0.00 | n.r. | 0.08 ± 0.00 | n.r. | 0.16 ± 0.02 |
| PCB BZ #170 | n.r. | n.r. | n.r. | n.r. | 0.30 ± 0.07 |
|  |  |  |  |  |  |
| *Polychloronaphthalenes (PCNs)* |  |  |  |  |  |
| MonoPCN, 2- | n.r. | 0.10 ± 0.00 | n.r. | n.r. | n.r. |
| **Compounds** | **Lake Tvillingvatnet** | **Lake Storvatnet** | **Lake Knudsenheia** | **Lake Glacier** | **Lake Solvannet** |
| S TetraPCN 1235 + 1256 | n.r. | n.r. | n.r. | n.r. | 0.10 ± 0.03 |
|  |  |  |  |  |  |
| ΣPAHs | 685.74 ± 3.54 | 103.50 ± 0.86 | 51.64 ± 0.86 | 18.55 ± 0.13 | 318.19 ± 3.11 |
| ΣCBs | 3.47 ± 0.01 | 3.20 ± 0.01 | 3.31 ± 0.02 | 3.08 ± 0.03 | 3.38 ± 0.02 |
| ΣPCBs | 0.66 ± 0.01 | 0.40 ± 0.01 | 0.53 ± 0.00 | 0.15 ± 0.01 | 2.27 ± 0.03 |
| ΣPCNs | n.r. | 0.10 ± 0.00 | n.r. | n.r. | 0.10 ± 0.03 |

b)

| **Compounds** | **Lake Sofia** | **Lake Argentina** | **Lake Telefon** | **Lake Ballaneros** | **Lake Zapatilla** | **Lake Crater** | **Lake Extremadura** |
| --- | --- | --- | --- | --- | --- | --- | --- |
| *Polycyclic Aromatic Hydrocarbons (PAHs)* |  |  |  |  |  |  |  |
| Naphthalene | 0.40 ± 0.00 | 0.40 ± 0.00 | 0.49 ± 0.00 | 1.06 ± 0.01 | 0.61 ± 0.00 | 0.589 ± 0.001 | 0.63 ± 0.00 |
| 2-methyl-naphthalene | 0.55 ± 0.00 | 0.53 ± 0.01 | 0.62 ± 0.01 | 1.02 ± 0.01 | 0.54 ± 0.00 | 0.521 ± 0.005 | 0.55 ± 0.00 |
| Acenaphthylene | 0.14 ± 0.00 | 0.14 ± 0.00 | 0.14 ± 0.00 | 0.32 ± 0.00 | 0.31 ± 0.00 | 0.300 ± 0.0002 | 0.32 ± 0.00 |
| Acenaphthene | 0.14 ± 0.00 | 0.17 ± 0.00 | 0.14 ± 0.00 | 0.27 ± 0.01 | 0.25 ± 0.00 | 0.262 ± 0.001 | 0.26 ± 0.00 |
| Fluorene | 0.19 ± 0.00 | 0.17 ± 0.00 | 0.19 ± 0.00 | 0.31 ± 0.00 | 0.20 ± 0.00 | 0.216 ± 0.001 | 0.23 ± 0.00 |
| Phenanthrene | 0.36 ± 0.01 | 0.37± 0.00 | 0.38 ± 0.01 | 1.14 ± 0.01 | 0.36 ± 0.00 | 0.289 ± 0.0003 | 0.41 ± 0.01 |
| Anthracene | 0.09 ± 0.00 | 0.09 ± 0.00 | 0.10 ± 0.00 | 0.19 ± 0.00 | 0.09 ± 0.00 | 0.072 ± 0.001 | 0.09 ± 0.00 |
| Fluoranthene | 0.23 ± 0.00 | 0.22 ± 0.00 | 0.22 ± 0.01 | 0.98 ± 0.01 | 0.17 ± 0.01 | 0.144 ± 0.001 | 0.19 ± 0.00 |
| **Compounds** | **Lake Sofia** | **Lake Argentina** | **Lake Telefon** | **Lake Ballaneros** | **Lake Zapatilla** | **Lake Crater** | **Lake Extremadura** |
| Pyrene | 0.22 ± 0.00 | 0.22 ± 0.00 | 0.27 ± 0.00 | 0.73 ± 0.01 | 0.19 ± 0.01 | 0.163 ± 0.002 | 0.19 ± 0.00 |
| Benz[a]anthracene | 0.47 ± 0.02 | 0.92 ± 0.01 | n.r. | 0.13 ± 0.00 | n.r. | n.r. | n.r. |
| Chysene | 1.19 ± 0.01 | 1.14 ± 0.01 | 0.83 ± 0.02 | 0.69 ± 0.00 | n.r. | n.r. | n.r. |
|  |  |  |  |  |  |  |  |
| *Chlorobenzenes (CBs)* |  |  |  |  |  |  |  |
| MonoCB | 0.36 ± 0.01 | 0.48 ± 0.01 | 0.56 ± 0.02 | 0.72 ± 0.04 | 0.44 ± 0.01 | 1.93 ± 0.04 | 0.43 ± 0.01 |
| DiCB 1,3- | 0.18 ± 0.00 | 0.18 ± 0.00 | 0.18 ± 0.00 | 0.18 ± 0.00 | 0.17 ± 0.00 | 0.169 ± 0.002 | 0.18 ± 0.01 |
| DiCB 1,4- | 0.25 ± 0.01 | 0.27 ± 0.01 | 0.28 ± 0.00 | 0.30 ± 0.01 | 0.24 ± 0.00 | 0.345 ± 0.007 | 0.24 ± 0.01 |
| DiCB 1,2- | 0.20 ± 0.00 | 0.20 ± 0.00 | 0.21 ± 0.00 | 0.21 ± 0.00 | 0.19 ± 0.00 | 0.194 ± 0.003 | 0.19 ± 0.01 |
| TriCB 1,3,5- | 0.17 ± 0.00 | 0.16 ± 0.00 | 0.17 ± 0.00 | 0.16 ± 0.00 | 0.15 ± 0.00 | 0.134 ± 0.002 | 0.16 ± 0.01 |
| TriCB 1,2,4- | 0.24 ± 0.00 | 0.23 ± 0.00 | 0.24 ± 0.00 | 0.24 ± 0.00 | 0.24 ± 0.00 | 0.221 ± 0.001 | 0.24 ± 0.00 |
| TriCB 1,2,3- | n.r. | n.r. | n.r. | 0.17 ± 0.00 | 0.16 ± 0.00 | 0.142 ± 0.001 | 0.17 ± 0.01 |
| TetraCB 1245 + 1235 | 0.17 ± 0.00 | 0.16 ± 0.00 | 0.16 ± 0.00 | 0.16 ± 0.00 | 0.15 ± 0.00 | 0.131 ± 0.004 | 0.15 ± 0.01 |
| TetraCB 1,2,3,4- | 0.16 ± 0.00 | 0.16 ± 0.00 | 0.16 ± 0.00 | 0.16 ± 0.00 | 0.14 ± 0.00 | 0.128 ± 0.003 | 0.15 ± 0.01 |
| PentaCB | n.r. | n.r. | n.r. | 0.08 ± 0.00 | 0.14 ± 0.00 | 0.064 ± 0.001 | 0.07 ± 0.00 |
| HexaCB | 0.15 ± 0.00 | 0.15 ± 0.00 | 0.15 ± 0.00 | 0.16 ± 0.00 | 0.14 ± 0.00 | 0.120 ± 0.002 | 0.15 ± 0.00 |
|  |  |  |  |  |  |  |  |
| *Polychlorobiphenyls (PCBs)* |  |  |  |  |  |  |  |
| S PCB BZ# 31 + BZ# 28 | 0.09 ± 0.00 | 0.09 ± 0.00 | 0.09 ± 0.00 | 0.09 ± 0.00 | 0.08 ± 0.00 | 0.074 ± 0.001 | 0.09 ± 0.00 |
| PCB BZ #52 | 0.10 ± 0.00 | 0.10 ± 0.00 | 0.10 ± 0.00 | 0.10 ± 0.00 | 0.09 ± 0.00 | 0.080 ± 0.001 | 0.09 ± 0.00 |
| PCB BZ #95 | 0.10 ± 0.00 | 0.10 ± 0.00 | 0.10 ± 0.00 | 0.10 ± 0.00 | 0.09 ± 0.00 | 0.079 ± 0.001 | 0.09 ± 0.00 |
| S PCB BZ# 84 + BZ# 90 + BZ# 101 | 0.10 ± 0.00 | 0.09 ± 0.00 | 0.09 ± 0.00 | 0.09 ± 0.00 | 0.08 ± 0.00 | 0.076 ± 0.001 | 0.09 ± 0.00 |
| **Compounds** | **Lake Sofia** | **Lake Argentina** | **Lake Telefon** | **Lake Ballaneros** | **Lake Zapatilla** | **Lake Crater** | **Lake Extremadura** |
| PCB BZ #99 | n.q. | 0.11 ± 0.00 | 0.11 ± 0.00 | 0.10 ± 0.00 | 0.09 ± 0.00 | n.r. | 0.10 ± 0.00 |
| PCB BZ #81 | n.r. | n.r. | n.r. | 0.09 ± 0.00 | 0.08 ± 0.00 | 0.075 ± 0.001 | 0.09 ± 0.00 |
| PCB BZ #110 | 0.10 ± 0.00 | 0.10 ± 0.00 | 0.10 ± 0.00 | 0.10 ± 0.00 | 0.09 ± 0.00 | 0.081 ± 0.001 | 0.09 ± 0.00 |
| PCB BZ #77 | n.r. | n.r. | n.r. | n.r. | n.r. | n.q. | n.r. |
| PCB BZ #151 | 0.08 ± 0.00 | 0.08 ± 0.00 | 0.08 ± 0.00 | 0.07 ± 0.00 | n.q. ± 0.00 | 0.060 ± 0.003 | n.q. |
| PCB BZ #149 | 0.08 ± 0.00 | 0.08 ± 0.00 | 0.08 ± 0.00 | 0.07 ± 0.00 | 0.05 ± 0.00 | n.q. | 0.06 ± 0.00 |
| PCB BZ #123 | 0.13 ± 0.00 | 0.09 ± 0.00 | 0.09 ± 0.00 | 0.10 ± 0.00 | 0.09 ± 0.00 | n.r. | 0.09 ± 0.00 |
| PCB BZ #118 | 0.10 ± 0.00 | 0.09 ± 0.00 | 0.10 ± 0.00 | 0.10 ± 0.00 | 0.09 ± 0.00 | 0.078 ± 0.001 | 0.10 ± 0.00 |
| S PCB BZ# 153 + BZ# 168 | 0.08 ± 0.00 | 0.07 ± 0.00 | 0.07 ± 0.00 | 0.07 ± 0.00 | 0.05 ± 0.00 | 0.056 ± 0.002 | 0.06 ± 0.00 |
| PCB BZ #105 | 0.08 ± 0.00 | n.r. | 0.09 ± 0.00 | n.r. | n.q. ± 0.00 | n.r. | n.r. |
| S PCB BZ# 138 + BZ# 158 | 0.07 ± 0.00 | 0.06 ± 0.00 | 0.07 ± 0.00 | 0.06 ± 0.00 | n.q. ± 0.00 | n.q. | n.q. ± 0.00 |
| S PCB BZ# 180 + BZ# 193 | n.r. | n.r. | n.r. | n.r. | 0.15 ± 0.00 | n.r. | 0.12 ± 0.00 |
|  |  |  |  |  |  |  |  |
| *Polychloronaphthalenes (PCNs)* |  |  |  |  |  |  |  |
| MonoPCN, 2- | n.r. | 0.08 ± 0.00 | 0.10 ± 0.00 | 0.13 ± 0.00 | 0.07 ± 0.00 | 0.066 ± 0.001 | 0.08 ± 0.00 |
| S TetraPCN 1235 + 1256 | 0.13 ± 0.00 | 0.12 ± 0.00 | 0.09 ± 0.00 | 0.14 ± 0.01 | 0.09 ± 0.00 | n.q. | n.r. |
| PentaPCN, 1,2,3,5,8- | 0.10 ± 0.00 | 0.09 ± 0.00 | 0.10 ± 0.00 | 0.09 ± 0.00 | 0.08 ± 0.00 | 0.068 ± 0.001 | 0.08 ± 0.00 |
| HexaPCN, 1,2,3,5,7,8- | n.r. | n.r. | n.r. | 0.19 ± 0.00 | n.r. | n.r. | n.r. |
| OctaPCN | n.r. | n.r. | n.r. | 2.31 ± 0.13 | 1.36 ± 0.13 | 2.56 ± 0.39 | 1.88 ± 0.30 |
|  |  |  |  |  |  |  |  |
|  |  |  |  |  |  |  |  |
|  |  |  |  |  |  |  |  |
| **Compounds** | **Lake Sofia** | **Lake Argentina** | **Lake Telefon** | **Lake Ballaneros** | **Lake Zapatilla** | **Lake Crater** | **Lake Extremadura** |
| ΣPAHs | 3.83 ± 0.01 | 4.35 ± 0.00 | 3.08 ± 0.01 | 6.29 ± 0.00 | 2.72 ± 0.00 | 2.56 ± 0.00 | 2.86 ± 0.00 |
| ΣCBs | 1.88 ± 0.00 | 1.99 ± 0.00 | 2.10 ± 0.00 | 2.49 ± 0.00 | 2.15 ± 0.00 | 3.58 ± 0.02 | 2.09 ± 0.01 |
| ΣPCBs | 0.95 ± 0.00 | 0.90 ± 0.00 | 0.96 ± 0.00 | 0.94 ± 0.00 | 1.11 ± 0.00 | 1.38 ± 0.00 | 0.87 ± 0.00 |
| ΣPCNs | 0.22 ± 0.00 | 0.24 ± 0.00 | 0.29 ± 0.00 | 2.73 ± 0.01 | 1.60 ± 0.04 | 2.69 ± 0.06 | 2.04 ± 0.10 |

c)

| Compounds | **Lake Solvannet** | **Lake Glacier** | **Lake Knudsenheia** | **Lake Storvatnet** | **Lake Tvillingvatnet** | **Lake Sofia** | **Lake Argentina** | **Lake Crater** | **Lake Zapatilla** | **Lake Extremadura** | **Lake Telefon** | **Lake Ballaneros** |
| --- | --- | --- | --- | --- | --- | --- | --- | --- | --- | --- | --- | --- |
| *Heavy Metals* |  |  |  |  |  |  |  |  |  |  |  |  |
| 7 Li | 7.9 | 12.6 | 24.8 | 12 | 18.5 | 3 | 5.4 | 2.5 | 2.05 | 1.56 | 2.5 | 1.04 |
| 9 Be | 0.42 | 0.39 | 1.32 | 0.6 | 1.1 | 0.23 | 0.45 | 0.15 | 0.17 | 0.1 | 0.13 | 0.13 |
| 51 V | 14.5 | 17.5 | 51 | 25 | 39 | 36 | 36 | 42 | 47 | 40 | 31 | 25 |
| 52 Cr | 13 | 23 | 37 | 20 | 31 | 6.3 | 9.2 | 2.4 | 4.6 | 7.2 | 4.1 | 1.9 |
| 55 Mn | 122.1 | 119.7 | 236.3 | 246.3 | 760.1 | 293.4 | 349 | 266.9 | 251.4 | 202.4 | 141.5 | 274.5 |
| 59 Co | 3.4 | 2.8 | 7.9 | 4.9 | 12.7 | 7.1 | 7.7 | 7.1 | 6.5 | 6 | 4.4 | 4.9 |
| 60 Ni | 7.8 | 14.1 | 22 | 11.4 | 24 | 7.8 | 9.9 | 5.2 | 5.3 | 7.5 | 6.5 | 3.2 |
| 63 Cu | 10.7 | 5.8 | 12.3 | 7.3 | 13.4 | 19.7 | 19.1 | 14.1 | 10.7 | 6.8 | 6.3 | 4.5 |
| 66 Zn | 103 | 26 | 56 | 38 | 61 | 30 | 56 | 18.4 | 14.5 | 14.8 | 10.6 | 9.4 |
| 75 As | 1.54 | 2.9 | 4.9 | 4 | 5.7 | 2.5 | 2.8 | 0.41 | 0.89 | 7.2 | 88 | 196 |
| 78 Se | 1.29 | 1.02 | 1.55 | 1.36 | 1.68 | 0.43 | 0.49 | 0.22 | 0.29 | 0.23 | 0.25 | 0.2 |
| 88 Sr | 41 | 75 | 52 | 23 | 27 | 24 | 22 | 21 | 27 | 31 | 36 | 31 |
| 95 Mo | 0.39 | 1.5 | 3.95 | 0.68 | 0.94 | 0.12 | 0.09 | 0.08 | 0.08 | 0.16 | 0.32 | 0.32 |
| 111 Cd | 0.29 | 0.31 | 0.23 | 0.13 | 0.43 | 0.07 | 0.14 | <0.04 | <0.04 | <0.04 | <0.04 | <0.04 |
| 121 Sb | 0.13 | 0.16 | 0.29 | 0.11 | 0.19 | 0.04 | 0.06 | <0.02 | <0.02 | 0.03 | 0.01 | 0.03 |
| 137 Ba | 50 | 33 | 96 | 53 | 101 | 27 | 28 | 7.8 | 8 | 5.7 | 8.5 | 8.7 |
| 205 Tl | 0.14 | 0.14 | 0.35 | 0.18 | 0.33 | 0.05 | 0.06 | 0.01 | 0.01 | 0.02 | 0.01 | 0.02 |
| 208 Pb | 7.8 | 4.1 | 13.9 | 5.8 | 13 | 12.2 | 24 | 0.69 | 0.51 | 0.4 | 1.36 | 2.01 |
| 238 U | 0.65 | 1.98 | 1.96 | 1.21 | 1.61 | 0.17 | 0.28 | 0.06 | 0.07 | 0.05 | 0.06 | 0.09 |
|  |  |  |  |  |  |  |  |  |  |  |  |  |
| Σ HM | 386 | 341 | 625 | 455 | 1113 | 470 | 572 | 388 | 379 | 331 | 342 | 563 |
